# Unusual Association of Large Cell Neuroendocrine Carcinoma of the Bladder and Prostatic Adenocarcinoma Within a 76-Year-Old Man

**DOI:** 10.7759/cureus.67651

**Published:** 2024-08-24

**Authors:** Karich Nassira, Mahmoud Aberkane, Anouar El Moudane, Anass Haloui, Amal Bennani

**Affiliations:** 1 Pathology, Mohammed VI University International Hospital, Oujda, MAR; 2 Pathology and Laboratory Medicine, Faculty of Medicine and Pharmacy of Oujda, Oujda, MAR; 3 Urology, Mohammed VI University International Hospital, Oujda, MAR; 4 Pathology, Faculty of Medicine, Mohammed VI University International Hospital, Mohamed I University, Oujda, MAR; 5 Anatomopathology, Faculty of Medicine and Pharmacy of Oujda, Oujda, MAR

**Keywords:** surgery., prostatic adenocarcinoma, urinary tumor, large cell neuroendocrine carcinoma, bladder cancer

## Abstract

Large cell neuroendocrine carcinoma (LCNEC) is one of the rarest types of bladder cancer, with an aggressive course and a poor prognosis. We report a case of LCNEC of the bladder associated with a prostatic adenocarcinoma. A very rare association, to our knowledge, is described for the first time in the literature. The patient was a 76-year-old non-smoker male, who presented with intermittent hematuria and dysuria. Cystoscopy revealed a lesion on the right lateral bladder wall. Biopsy was in favor of a LCNEC with muscle invasion. The CT scan showed the presence of a second lesion in the prostate, confirmed by histological examination. The patient was treated by neoadjuvant chemotherapy of the cisplatin-etoposide type for large-cell bladder neuroendocrine carcinoma, and hormone therapy with luteinizing hormone-releasing hormone (LH-RH) analogs for prostatic adenocarcinoma, followed by radical surgery. Given the rarity of this tumor, LCNEC treatment lacks precision. Many cases are published with different therapeutic strategies. A literature review would be interesting to codify the therapeutic strategy for this rare tumor.

## Introduction

Bladder cancer (BC) is the tenth most commonly diagnosed cancer worldwide [1]. Its annual incidence increased from 4,30,000 cases in 2012 to 5,50,000 in 2018 [2,3]. Urothelial carcinoma is the predominant histologic type. According to the World Health Organization classiﬁcation, it represents more than 90% of BC and around 5% are squamous cell carcinoma [3]. Neuroendocrine tumors are generally rarely described in this location. Even more so, large cell neuroendocrine carcinomas are rarer than small cell neuroendocrine carcinomas (SCNECs). What makes our case special is the association with prostatic adenocarcinoma, a situation never described in the literature. All this makes the management of this patient special and debatable.

## Case presentation

**Figure 1:**
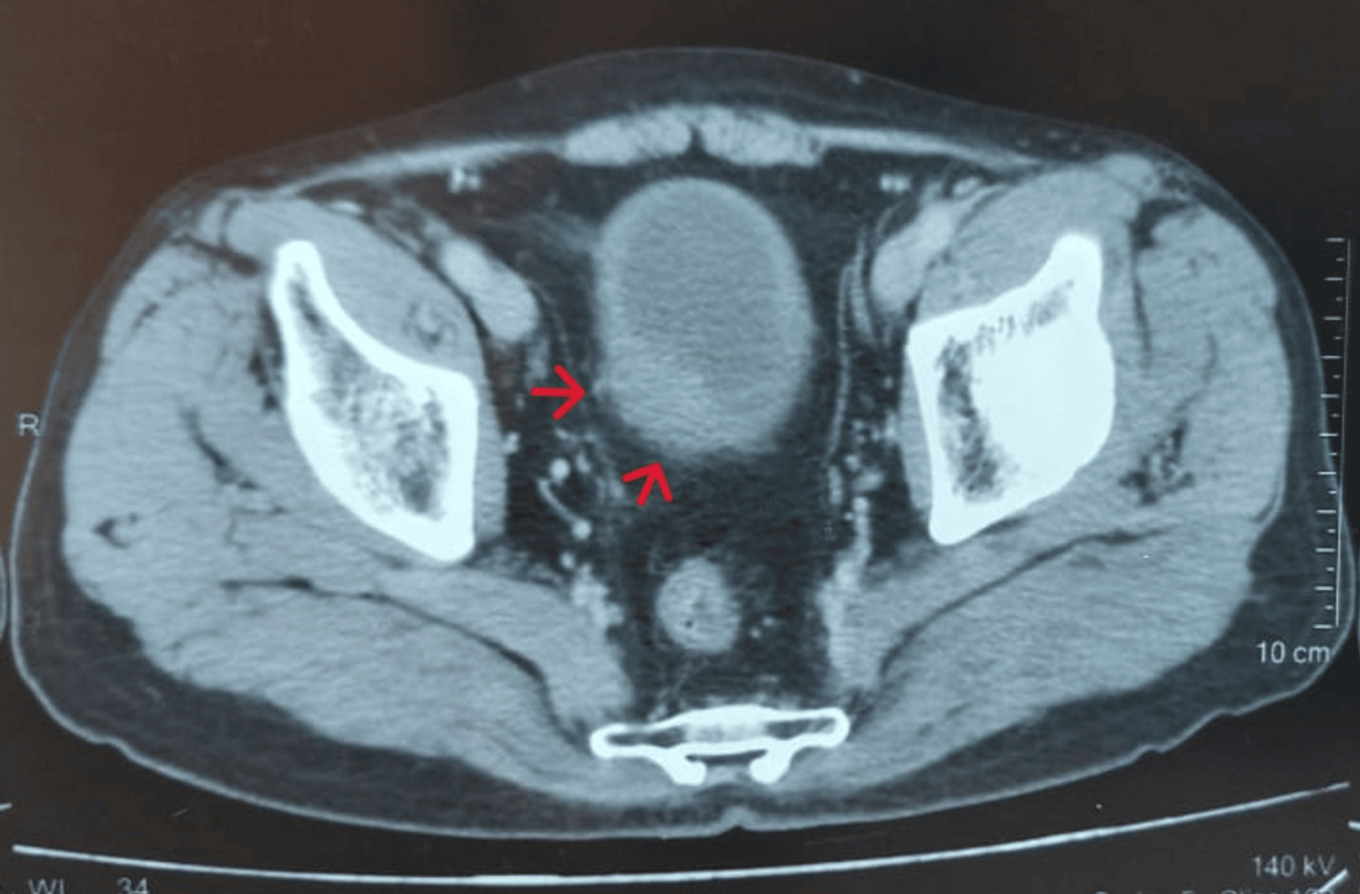
The CT scan showed a circumferential tumoral lesion of the bladder with poorly defined margins and infiltration of peri vesical fat (red arrows).

We report the case of a 76-year-old nonsmoking man without significant past medical history. Presented with intermittent hematuria and dysuria. He underwent a cystoscopy that revealed a lesion on the right lateral bladder wall, measuring 22 mm in diameter. A transurethral resection (TURB) was performed, and histological examination was in favor of a large cell neuroendocrine carcinoma (LCNEC) with muscle invasion. Computed tomography of the brain, cervical, chest, abdomen, and pelvis showed a circumferential tumoral lesion of the bladder with poorly defined margins and infiltration of peri vesical fat (Figure 1). In addition, the CT scan revealed a hypervascular lesion in the peripheral zone of the prostate, measuring 18 mm in the long axis, highly suspicious of malignancy. An MRI scan confirmed the presence of a peripheral zone prostate lesion, classified PIRADS 4, confined to the prostate gland. Subsequently, the patient underwent a prostate biopsy. The anatomopathological result showed an adenocarcinoma of the right lobe of the prostate with a modified Gleason score of 7(3+4), deemed intermediate risk.

In view of these findings, the patient was discussed at a multidisciplinary consultation meeting, and a decision was taken to opt for neoadjuvant chemotherapy of the cisplatin-etoposide type for large-cell bladder neuroendocrine carcinoma, and hormone therapy with luteinizing hormone-releasing hormone (LH-RH) analogs for prostatic adenocarcinoma.

After four cycles of chemotherapy, a follow-up CT scan showed a significant reduction in the size of the bladder lesion to 12 mm without infiltration of the peri-bladder fat. The prostate lesion was no longer visible. Immediately afterward, the patient was scheduled for radical cystoprostatectomy with curage.

**Figure 2:**
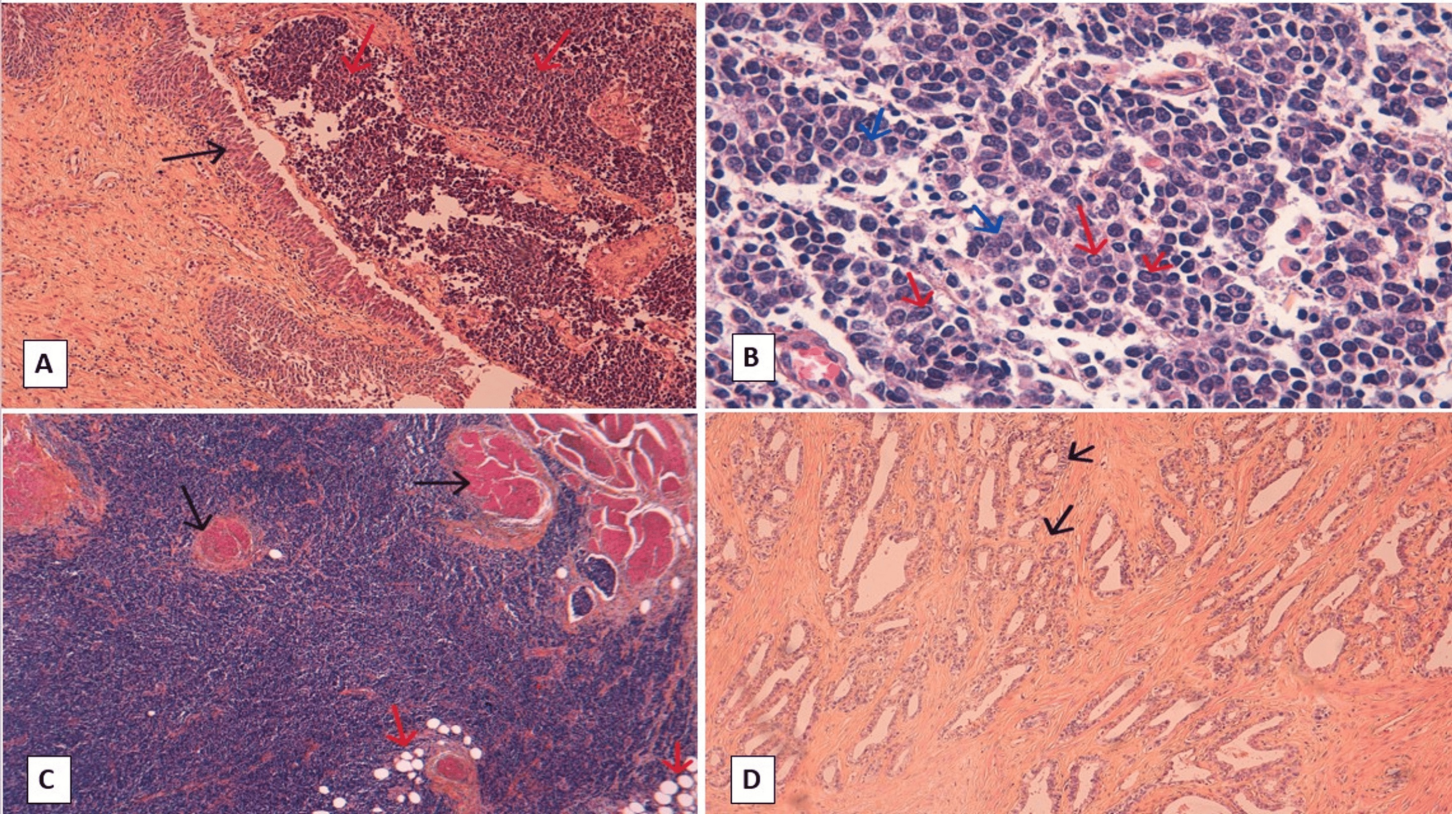
Histology: (A) Micrograph showing bladder mucosa (black arrow) infiltrated by sheet-like proliferation (red arrows) (HEx100). (B) At higher magnification (HEx400), the tumor is made of polygonal cells with dusty chromatin (blue arrows) showing prominent nucleoli (red arrows). (C) Tumor infiltrates bladder muscle (black arrows) and perivesical fat (red arrows) (HEx100). (D) Glandular structures (black arrows) correspond to prostatic adenocarcinoma.

**Figure 3:**
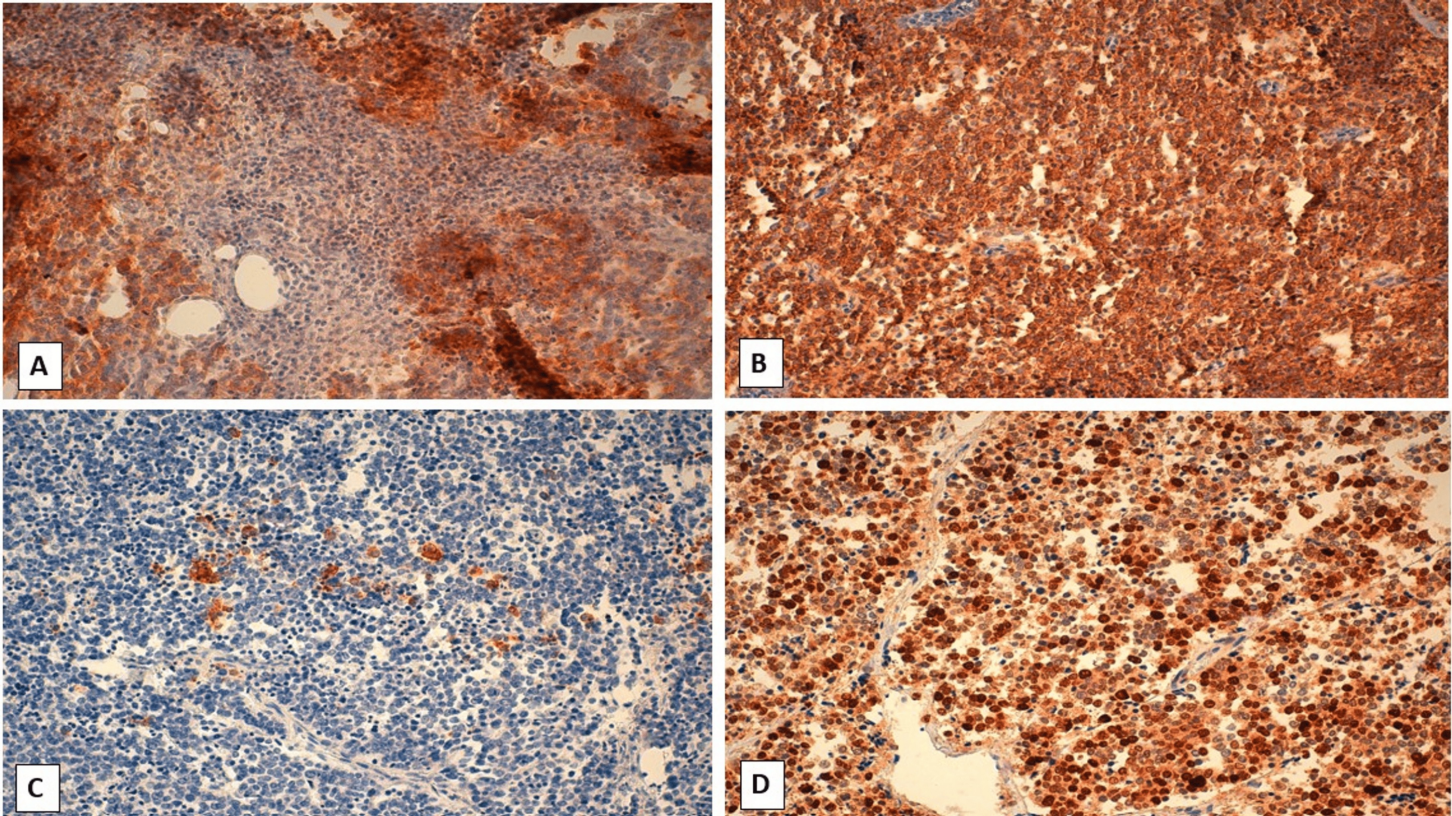
Immunohistochemistry: (A) strong labeling of tumor cells with synaptophysin (HEx100), (B) strong, diffuse CD56 labeling of tumor cells (HEx100), (C) weak, heterogeneous staining of tumor cells by chromogranin (HEx100), and (D) tumor cells show diffuse Ki67 staining (HEx100).

A histological study of the bladder lesion revealed tumor proliferation organized in diffuse sheets, with clear cytonuclear atypia and granular, dusty chromatin (Figure 2). The immunohistochemical study confirmed the neuroendocrine nature of the tumor cells, which diffusely expressed synaptophysin and CD 56, and focally chromogranin A and pan-cytokeratin (CK). The cell proliferation index (Ki67) was estimated at 80% (Figure 3). Tumor cells invade the bladder muscle and peri-bladder fat, without invading the prostate gland. On the other hand, histological examination of the prostate shows a tumor proliferation made up of well-differentiated glands (Gleason score 6(3+3)) occupying 20% of the tumor volume and confined to the prostate gland. An immunohistochemical study confirmed the tumoral origin of the glandular proliferation in the prostate.

Post-op, for the neuroendocrine tumor, we added two cycles of chemotherapy (cisplatin-etoposide). And for prostatic adenocarcinoma, we have completed six months of hormone therapy. Six months after surgery, the patient had pelvic metastatic adenopathy and was, therefore, undergoing palliative chemotherapy.

## Discussion

Bladder localization of neuroendocrine tumors is rare; the first description was reported in 1981 by Solcia et al. [4,5]. However, this organ remains the most affected in the urinary tract compared to the others (prostate, kidney, ureter) [4,6]. LCNEC is an aggressive histological type, most often presenting with muscle invasion (88%) and metastases (30%). It affects men more than women (3x). Elderly patients are most frequently concerned, with a median age of 63 years [7]. About 67% of patients are smokers, which was not the case for our patient. The clinical presentation is dominated by hematuria [8]. The same finding was noted in this case. Cystoscopy often reveals polypoid lesions with necrotic and ulcerated surfaces, varying in size from 4 to 10 cm. The lateral wall of the bladder is the preferred location for large cell neuroendocrine carcinomas (54%) [6], which is consistent with our case.

LCNEC exhibits typical features of a neuroendocrine tumor, such as organoid nesting, palisading, rosettes, and trabecular patterns, as well as a high mitotic rate. Immunohistochemical staining is positive for neuroendocrine markers. However, it differs from small-cell neuroendocrine carcinoma mainly in its nuclear size and the presence of a prominent nucleolus [9].

The number of cases of large cell neuroendocrine carcinoma of the bladder described in the literature does not exceed 40 [10-15]. To our knowledge, no association with prostatic adenocarcinoma has been described. This makes this case very interesting.

At present, no guidelines have been published concerning the therapeutic modalities for LCNEC. Because it is a rare cancer, no prospective, randomized trials have been carried out. A multimodal therapeutic approach using cisplatin-based chemotherapy, radiotherapy, and partial or radical surgery has been reported in the majority of published cases [10,11,16,17]. Dowd et al. reported no recurrence at one year after the treatment by surgical resection and adjuvant chemotherapy and radiotherapy [11]. On top of this, another study by Niu et al. reported higher survival rates in patients treated by multimodal therapies compared to patients treated with a conservative treatment. This is the same therapeutic approach envisaged for our patient, in association with hormonal treatment for prostatic adenocarcinoma.

The majority of published studies report a pejorative prognosis for LCNEC, comparable with that of SCNEC [18-20]. In our case, the prognosis is obviously linked to the bladder lesion (LCNEC). Six months after surgery, the patient had pelvic metastatic adenopathy and was therefore undergoing palliative chemotherapy. As supported by the Wang et al. study, prompt and early diagnosis, followed by an appropriate therapeutic approach based on neoadjuvant chemotherapy, radical cystectomy may provide long-term control of a localized tumor with extended overall survival [20].

## Conclusions

Because of its rarity, the management of large cell neuroendocrine carcinoma of the bladder is based on cases reported in the literature. Its association with prostatic adenocarcinoma makes management more complex. Prospective studies are therefore recommended in order to develop codified therapeutic strategies.
